# Correlation between the progressive cytoplasmic expression of a novel small heat shock protein (Hsp16.2) and malignancy in brain tumors

**DOI:** 10.1186/1471-2407-7-233

**Published:** 2007-12-21

**Authors:** Eva Pozsgai, Eva Gomori, Andras Szigeti, Arpad Boronkai, Ferenc Gallyas, Balazs Sumegi, Szabolcs Bellyei

**Affiliations:** 1 Department of Biochemistry and Medical Chemistry, University of Pécs, Pécs, Hungary; 2 Department of Pathology, University of Pécs, Pécs, Hungary; 3 Department of Oncotherapy, University of Pécs, Pécs, Hungary

## Abstract

**Background:**

Small heat shock proteins are molecular chaperones that protect proteins against stress-induced aggregation. They have also been found to have anti-apoptotic activity and to play a part in the development of tumors. Recently, we identified a new small heat shock protein, Hsp16.2 which displayed increased expression in neuroectodermal tumors. Our aim was to investigate the expression of Hsp16.2 in different types of brain tumors and to correlate its expression with the histological grade of the tumor.

**Methods:**

Immunohistochemistry with a polyclonal antibody to Hsp16.2 was carried out on formalin-fixed, paraffin-wax-embedded sections using the streptavidin-biotin method. 91 samples were examined and their histological grade was defined. According to the intensity of Hsp16.2 immunoreactivity, low (+), moderate (++), high (+++) or none (-) scores were given.

Immunoblotting was carried out on 30 samples of brain tumors using SDS-polyacrylamide gel electrophoresis and Western-blotting.

**Results:**

Low grade (grades 1–2) brain tumors displayed low cytoplasmic Hsp16.2 immunoreactivity, grade 3 tumors showed moderate cytoplasmic staining, while high grade (grade 4) tumors exhibited intensive cytoplasmic Hsp16.2 staining. Immunoblotting supported the above mentioned results. Normal brain tissue acted as a negative control for the experiment, since the cytoplasm did not stain for Hsp16.2. There was a positive correlation between the level of Hsp16.2 expression and the level of anaplasia in different malignant tissue samples.

**Conclusion:**

Hsp16.2 expression was directly correlated with the histological grade of brain tumors, therefore Hsp16.2 may have relevance as becoming a possible tumor marker.

## Background

Most malignant neoplasms in the brain already carry a dismal prognosis when they are diagnosed. Therefore, the treatment of human brain tumors possesses a continuous challenge for oncological research. Although several brain tumor markers have been studied as possible prognostic factors, further study is needed to reveal the complex mechanism of tumor genesis, thus helping to discover the most appropriate tumor markers for prognosticating the neoplasm.

Small heat shock proteins have been noted for their possible role in the development of tumors [1,2]. Functionally these proteins are molecular chaperones that prevent the stress induced aggregation of denatured proteins, thus protecting protein function and activity[3,4]. Furthermore, sHSPs have anti-apoptotic activity, which -if overexpressed in tumor cells – could lead to increased tumor growth, a resistance to chemo- or radiotherapy and to the unfortunate outcome of the disease [5-8].

Previously we identified and characterized a novel small heat shock protein (Hsp16.2) [9]. Overexpression of Hsp16.2 protected cells against various stress stimuli (e.g. hydrogen peroxide, taxol) by the stabilization of the mitochondrial membrane system and the inhibition of caspase 3 activation. It was also demonstrated that the anti-apoptotic effect of Hsp16.2 is mediated by the activation of Hsp90, with which Hsp16.2 binds, and by the activation of the PI-3 kinase-Akt pathway. Furthermore, overexpression of Hsp16.2 increased lipid rafts formation, thus helping to stabilize the plasma membrane [9,10].

Preliminary studies indicated that Hsp16.2 is expressed in neuroectodermal tumors [9]. In the present paper we study the different types of brain tumors including benign and malignant meningeoma, oligodendroglioma, glioblastoma multiforme, ependymoma and medulloblastoma. Using immunohistochemistry method and Western blot analysis we determined the expression of Hsp16.2 and its intracellular localization in the tumor cells. Our aim was to examine whether the level of Hsp16.2 expression correlates with the malignancy of the brain tumor. This study provides evidence for the role of Hsp16.2 as a possible marker for brain tumors.

## Methods

### Tumor materials

Brain tumor samples from ninety-one patients were available for examination. All tumor tissue specimens were provided by the Medical University of Pécs, Department of Neurosurgery and Pathology. Full ethical approval was given by the Regional Research Ethics Committee of the Medical Center of Pécs (Approval Number: 030630). for the use of these samples. Each type of tumor was identified according to the revised WHO classification of Histological Typing of the Tumors of the Central Nervous System (2007) [11].

### Preparation of polyclonal antibodies against Hsp16.2

Rabbits were immunized subcutaneously at multiple sites with 100 pg of recombinant Hsp16.2/GST fusion protein which was expressed as it was described before [9,10] in Freund's complete adjuvant. Four subsequent booster injections at 4-week intervals were given with 50 pg of protein in Freund's incomplete adjuvant. Blood was collected 10 days after boosting, and the antiserums were stored at -20 C. IgGs were affinity purified from sera by protein G-Sepharose chromatography according to the manufacturer's protocol.

### Immunohistochemistry

Sections from the tumor tissue samples were formalin-fixed and paraffin-embedded. Subsequently, they were incubated with polyclonal anti-Hsp16.2 polyclonal antibody. Immunohistochemical staining was carried out according to the streptavidin-biotin-peroxidase method with hydrogen peroxide/3-amino-9-ethylcarbazole development using the Universal kit. Only secondary IgG was incubated with the control sections. The evaluation of the slides was done with the help of an Olympus BX50 light microscope with incorporated photography system (Olympus Optical Co., Hamburg, Germany). Both the presence and localization of positive staining for Hsp16.2 was examined. Staining intensity was recorded semiquantitatively as mild (+), moderate (++) or strong (+++), following as it was described before [12]. For internal positive control, the normal cerebral and vascular structures of the samples were used. Positive areas around necrotic fields were excluded due to their probable stress related up-regulation.

### Immunoblot analysis

Tumor tissue specimens were homogenized in chilled lysis buffer of 0,5 mM sodium metavanadate, 1 mM EDTA, and protease inhibitor mixture in phosphate-buffered saline in a Teflon/glass homogenizer, and centrifuged. Isolation of cytosol and nuclear fractions were carried out by standard lab protocols exactly as previously [13]. The samples were equalized to 1 mg/ml total protein concentration using Biuret's method and subjected to SDS-PAGE. Proteins (20 μg/lane) were separated on 15% gels and then transferred to nitrocellulose membranes. The membranes were blocked in 5% low fat milk for 1 h at room temperature, then exposed to the primary anti-Hsp16,2 antibodies at 4°C overnight at a dilution of 1:2,000 in blocking solution. Appropriate horseradish peroxidase-conjugated secondary antibodies were used for 2 h at room temperature and at 1:5,000 dilution. Peroxidase labeling was visualized with enhanced chemiluminescence (ECL) using an ECL Western blotting detection system (Amersham Biosciences). The developed films were scanned, and the pixel volumes of the bands were determined using NIH Image J software. All experiments were repeated four times.

### Statistical analysis

Difference in distribution of variables between groups was tested using χ^2 ^test. Values of p < 0.01 were considered to be significant.

## Results

### Expression and intracellular localization of Hsp16.2 in different brain tumors by immunohistochemistry

Ninety-one samples of different brain tumors were evaluated in the present study (Table 1): 5 schwannomas (grade 1), 6 pilocytic astrocytomas (grade 1), 6 meningothelial meningeomas (grade 1), 5 fibrous meningeomas (grade 1), 8 diffuse astrocytomas (grade 2), 5 oligodendrogliomas (grade 2), 6 ependymomas (grade 2), 5 atypical meningeomas (grade 2), 6 malignant meningeomas (grade 3), 5 anaplastic astrocytomas (grade 3), 5 anaplastic oligodendrogliomas (grade 3), 9 glioblastomas (grade 4), 5 giant cell glioblastomas (grade 4), 8 medulloblastomas (grade 4) and 7 PNETs (primitive neuroectodermal tumor) (grade 4).

**Table 1:** Immunohistochemical analysis of Hsp16.2 in 51 human brain tumors.

Histological diagnosis	No. of cases	Tumor grade	Intracytoplasmic labeling				Intranuclear labeling			
			
			-	+	++	+++	-	+	++	+++
Schwannoma	5	1	5							5
Pilocytic astrocytoma	6	1	3	3						6
Meningothelial meningioma	6	1	4	2						6
Fibrous meningeoma	5	1	2	3						5
Diffuse astrocytoma	8	2	3	5						8
Oligodendroglioma	5	2	1	4						5
Ependymoma	6	2		1	5				1	5
Atypical meningeoma	5	2		1	4					5
Malignant meningeoma	6	3			6					6
Anaplastic astrocytoma	5	3			5					5
Anaplastic oligodendroglioma	5	3			5					5
Glioblastoma	9	4			2	7				9
Giant cell glioblastoma	5	4				5				5
Medulloblastoma	8	4				8				8
PNET	7	4				7				7

Staining intensity: (+) mild, (++) moderate, (+++) strong

**Figure 1:**
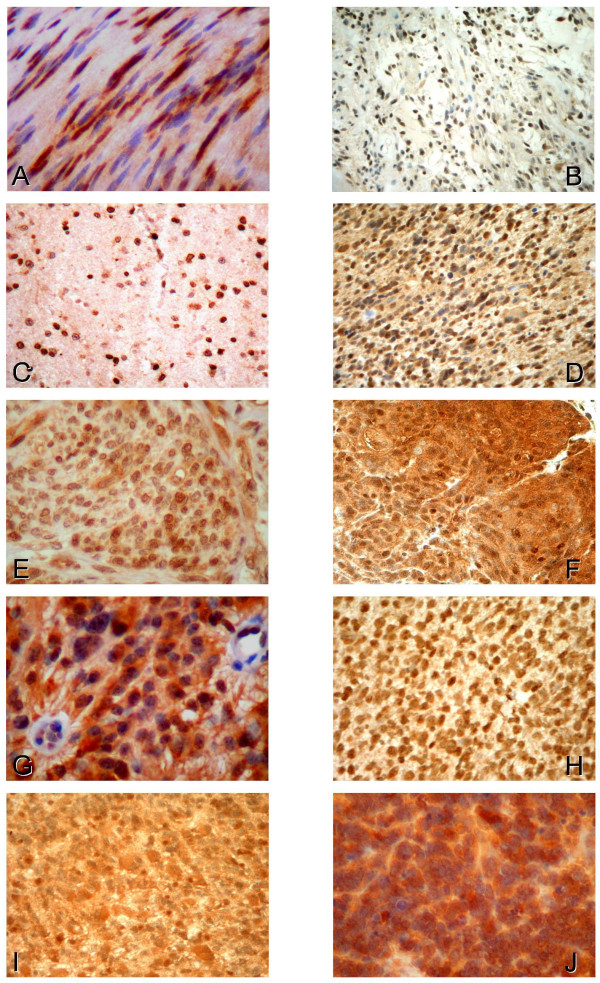
**Expression and intracellular distribution of Hsp16.2 in different human tumors of the nervous system**. Immunohistochemistry utilizing anti-Hsp16.2 primary antibody was performed on 91 brain tumor samples. **A**. Schwannoma. Intensive intranuclear Hsp16.2 immunoreactivity, whereas no immunoreactivity in the cytoplasm. **B**. Pilocytic astrocytoma. Strong intranuclear immunopositivity but no intracytoplasmic staining was detected. **C**. Grade 2 astrocytoma shows intensive intranuclear labeling as well as mild cytoplasmic staining. **D**. Grade 3 astrocytoma exhibits strong Hsp 16.2 positivity intranuclearly and moderate Hsp16.2 positivity in the cytoplasm. **E. **Grade 1 meningeoma showing high expression of Hsp16.2 intranuclearly and mild expression in the cytoplasm. **F**. Grade 3 meningeoma displayed strong intranuclear and moderate cytoplasmic staining for Hsp16.2. **G. **Grade 2 ependymoma with strong intranuclear and moderate cytoplasmic immunopositivity. **H. **Grade 3 oligodendroglioma exhibiting intensive intranuclear and moderate cytoplasmic immunoreactivity. **I**. Grade 4 glioblastoma showing strong Hsp16.2 positivity intranuclearly and intracytoplasmically alike. **J**. Grade 4 PNET with intensive staining in the nucleus as well as in the cytoplasm.

Hsp16.2 immunoreactivity was found both in the nucleus and in some cases in the cytoplasm in tumor tissues (Fig. 1). Since intranuclear labeling was present in all tumor samples in large quantities, cytoplasmic Hsp16.2 immunoreactivity could be used for differential diagnostic purposes. Cytoplasmic labeling varied considerably among the different histological types and grades of tumors. Low grade tumors (grades 1–2) showed weak or no staining (+/-) in the cytoplasm (Fig. 1.A,B). There was no detectable Hsp16.2 in the cytoplasm of the benign schwannoma (Fig. 1.A) and one pilocytic- (Fig 1.B) and two diffuse astrocytomas. The remaining low grade tumors stained weakly (Fig. 1.C,E), excepting ependymomas (Fig. 1.G) and atypical meningeomas, which stained moderately (++).

Grade 3 tumors, including malignant meningeomas (Fig. 1.F), anaplastic astrocytomas (Fig 1.D) and oligodendrogliomas (Fig. 1.H), displayed moderate cytoplasmic immunoreactivity for Hsp16.2. (++). High grade tumors (grade 4) such as glioblastomas (Fig. 1.I), medulloblastomas and PNETs (Fig. 1.J) exhibited strong Hsp16.2 positivity in the cytoplasm. (+++)

Table 2 shows the correlation between the cytoplasmic staining and the histological grades of different brain tumors.

**Table 2:** Correlation of Hsp16.2 cytoplasmic expression and histological grade of brain cancer.

Hsp16.2 expression	Grade 1 n (%)	Grade 2 n (%)	Grade 3 n (%)	Grade 4 n (%)	Total n (%)	P Value
-	14 (63.6)	4 (16.6)			18 (19.8)	<0.01
+	8 (36.4)	11(45.8)			19 (20.8)	
++		9 (37.6)	16 (100)	2 (6.9)	27 (29.7)	
+++				27 (93.1)	27 (29.7)	
Total	22 (100)	24 (100)	16 (100)	29 (100)	91 (100)	

Staining intensity: (+) mild, (++) moderate, (+++) strong

There was no cytoplasmic expression in 63.6% and mild expression in 36.4% of Grade 1 cancers. 16.6 % showed no, 45.8% exhibited mild and 37.6% of the samples displayed moderate staining in Grade 2 cancers. All (100%) Grade 3 cancers demonstrated moderate staining. Only 6.9% of Grade 4 tumor samples revealed moderate immunoreactivity, while 93.1% proved to be intensively stained. These results clearly demonstrate that the Hsp16.2 staining of the cytoplasm is directly correlated with the histological grade of the brain tumors.

### Detection of Hsp16.2 expression by Western-blot

**Figure 2:**
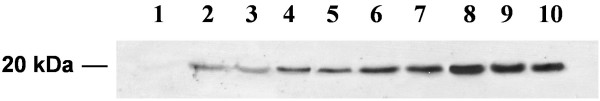
**Cytoplasmic expression of Hsp16.2 in different human brain tumors**. Endogenous cytoplasmic expression levels of Hsp16.2 were assessed by Western blotting utilizing a custom made polyclonal anti-Hsp16.2 primary antibody. 30 samples were used including three from normal brain tissue and 18 samples from nine different types of brain cancer, two samples from each type. The subcellulare fractionation was confirmed by probing with antibodies recognizing nuclear H3 histone, cytoplasmic actin and equal loading was confirmed by a second incubation with anti-GAPDH antibody (data not shown). 1: normal brain, 2: Pilocytic astrocytoma (Grade 1), 3: Meningothelial meningioma (Grade1) 4: Diffuse astrocytoma (Grade 2), 5: Ependymoma (Grade 2), 6: Malignant meningioma (Grade 3), 7: Anaplastic astrocytoma (Grade 3), 8: Medulloblastoma (Grade 4), 9: Giant cell glioblastoma (Grade 4), 10: PNET (Grade 4).

After subcellular fractionation, the expression of Hsp16.2 was determined from both the cytosolic (Fig. 2) and the nuclear fraction by immunoblotting. Thirty samples were studied including three from normal brain tissue, and three samples from nine different types of brain tumors. The intranuclear expression of Hsp16.2 was approximately the same level in the different samples (data not shown). Three samples from different parts of the brain were tumor-free, serving as a negative control for the experiment, which contained a minimal amount of nuclear and cytoplasmic Hsp16.2 (lane 1). Low cytoplasmic expressions of the protein were visible in grade 1 meningeomas, pilocytic astrocytomas (lane 2, 3). The expression was stronger in the Grade 2 diffuse astrocytomas and ependymomas (lane 4, 5). Higher cytoplasmic expressions of Hsp16.2 were observed in grade 3 tumors such as anaplastic astrocytomas and malignant meningeomas (6, 7). The strongest bands appeared in the cytoplasmic samples of medulloblastomas, PNETs and glioblastomas (8–10). In conclusion, the observed Hsp16.2 labeling correlated with the results gained by the immunohistochemistry method.

## Discussion

Small stress proteins are a group of heat shock proteins that share an evolutionary conserved C-terminal region, called the alpha-crystallin domain, and whose molecular weight ranges between 15 and 43 kDas [1,7,14-16]. They are known to play a part in cell differentiation and in counteracting apoptosis [7]. Due to the anti-apoptotic activity of sHsps, tumor cells expressing the protein highly may become increasingly resistant to chemo- or radiotherapy[1,17-19].

A number of recent studies have been concerned with the possible role of small heat shock proteins in the progression of brain tumors. Aoyama et al. reported increased expression of alphaB-crystallin in human glial tumors such as astrocytomas and glioblastoma multiforme[20]. Astrocytic neoplasms were examined for Hsp27 immunoreactivity also. While normal astrocytes showed no Hsp27 immunoreactivity, low Hsp27 expression was found in benign astrocytomas and elevated levels of Hsp27 found in poorly differentiated tumors. There are data indicating the direct correlation between Hsp27 expression and the histologic grade of astrocytic tumors [17,21-23]. The prognostic significance of Hsp27, Hsp 70, and Hsp90 in medulloblastomas was examined with equivocally positive results [24].

Previously, we reported the existence of a novel small stress protein, sHsp16.2 and its high levels of expression in neuroectodermal cancers [9]. This finding turned our attention to examining sHsp16.2 expression in brain tumors differing in their grade and type. Since all fifty-one tumors were labeled equally intra-nuclearly, and they varied in their cytoplasmic labeling, it became apparent that the various tumors differed in the distribution and density of sHsp16.2 in the cell cytoplasm. In accordance with earlier studies, astrocytomas (grade 1–2) as well as other benign brain tumors, such as meningothelial meningeoma, and oligodendroglioma (grade 1–2) exhibited low expression (+) of Hsp16.2. In fact, certain benign brain tumors, like schwannoma and fibrous meningeoma showed no immunoreactivity at all. However, the increase of anaplasia in the tumor cells resulted in moderate (++) expression (atypical meningeoma, malignant meningeoma, anaplastic astrocytoma, anaplastic oligodendroglioma) and high (+++) expression (glioblastoma, medulloblastoma, PNET) of the protein. Thus, it became evident that there is a direct correlation between the intensity of the staining and the histological grade of the brain tumor. Western blot analysis of thirty tumor samples gave similar results. It was observed, that no Hsp16.2 was found in normal brain tissue, while high Hsp16.2 labeling was observed in samples from medulloblastoma and glioblastoma. The observation that low levels were seen in Grade 1 meningeomas, pilocytic astrocytomas, moderate levels in grade 2 diffuse astrocytomas and ependynoma, stronger in grade 3 anaplastic astrocytomas and malignant melanoma, and elevated levels of Hsp16.2 in grade 4 neoplasms indicate that Hsp16.2 protein levels increase with the grade of the tumor.

According to our findings, Hsp16.2 could be a marker whose cytoplasmic expression increases as the tumor progresses. The cytoplasmic expression of the protein correlates directly with the grade of the tumors, it is only present in tumor cells in significant quantity and its level increases with the increase of cell anaplasia.

## Conclusion

We have found that the Hsp16.2 could become a valuable marker for primary brain tumor diagnosis and the anti-apoptotic activity of sHsp16.2 could become the target of drug therapy. Still, further efforts need to be made to reveal the exact role and function of sHsp16.2 in tumor cells. However, the significance of this novel small stress protein in anti-cancer research seems certain.

## List of abbreviations

sHsp, small heat shock protein; PNET, primitive neuroectodermal tumor

## Competing interests

The author(s) declare that they have no competing interests.

## Authors' contributions

EP carried out all of the molecular biological experiments and drafted the manuscript.

EG carried out the immunhistological studies

AS participated in the design of the study and performed the statistical analysis

AB participated in collecting the samples

FGJ has been involved in revising the manuscript

BS has been involved in revising the manuscript critically for important intellectual content

SB has been involved in designing the study, in the coordination of collecting the samples and drafting the manuscript

All authors read and approved the final manuscript.

## Pre-publication history

The pre-publication history for this paper can be accessed here:


